# Lemierre Syndrome

**DOI:** 10.5334/jbsr.2792

**Published:** 2022-05-04

**Authors:** Axel Boyer, Laurent Médart, Laurent Collignon

**Affiliations:** 1 Hopital de la citadelle – Liège, BE

**Keywords:** jugular thrombophlebitis, septic pulmonary emboli

## Abstract

**Teaching Point:** Lemierre syndrome is a rare complication of bacterial pharyngitis with cervical thrombophlebitis leading to pulmonary abcesses; the radiologist may have a key role.

## Case History

A 60-year-old male presented to the emergency unit with deterioration of general status for ten days, with non-productive cough. Physical exam revealed tachycardia, fever, low blood pressure, and desaturation. Blood test showed elevated CRP and leukocytosis, leading to the diagnostic of early septic shock.

**Figure 1:**
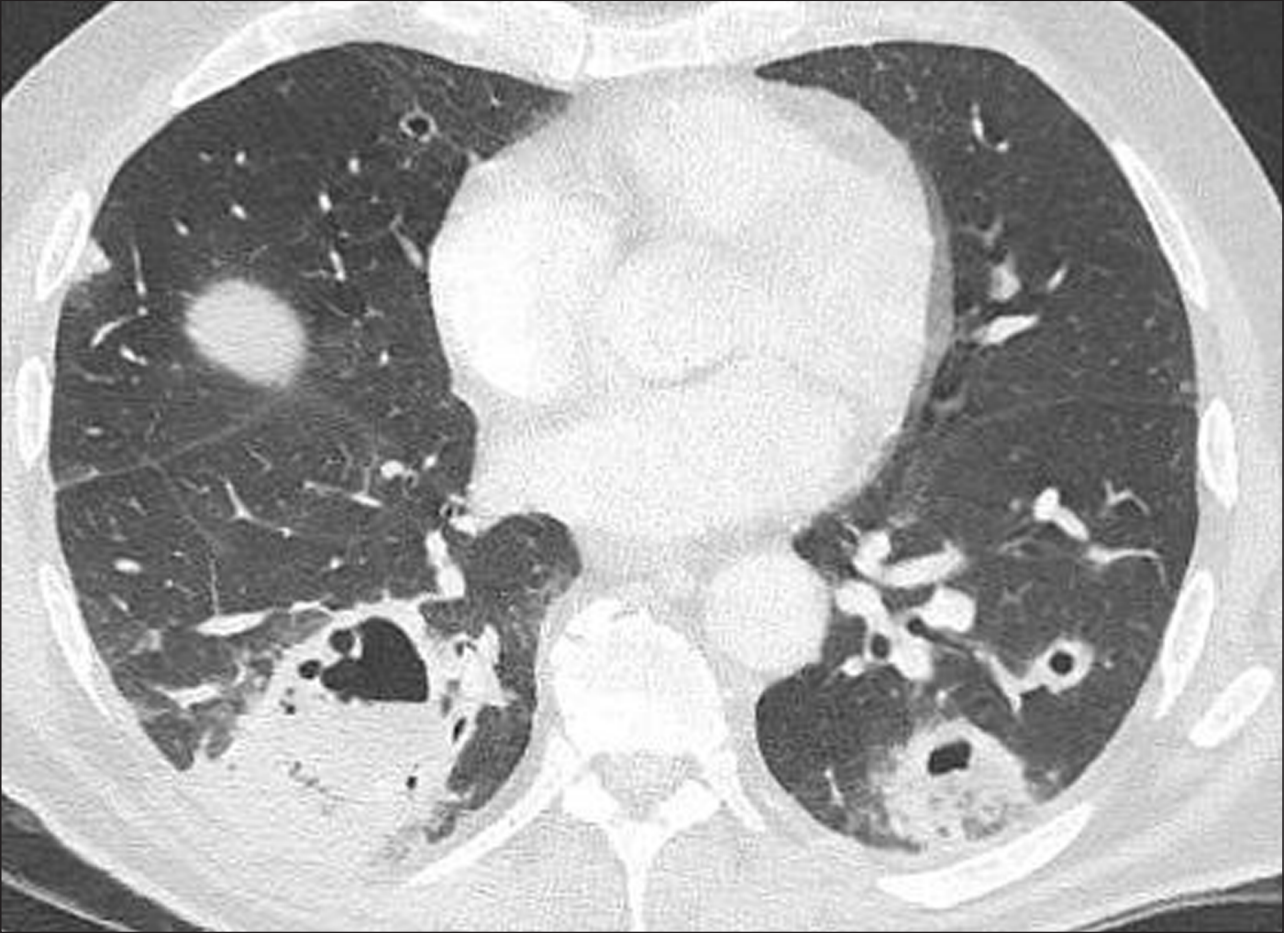


Contrast-enhanced computed tomography (CECT) of the thorax and abdomen depicted multiple excavated pulmonary nodules and masses consistent with abscesses (***Figure 1***), associated to hilar and mediastinal lymphadenopathies. Mass effect at the right base of the neck was suggestive of thrombophlebitis of the right internal jugular vein. The whole picture in septic context was strongly indicative for a Lemierre syndrome.

**Figure 2:**
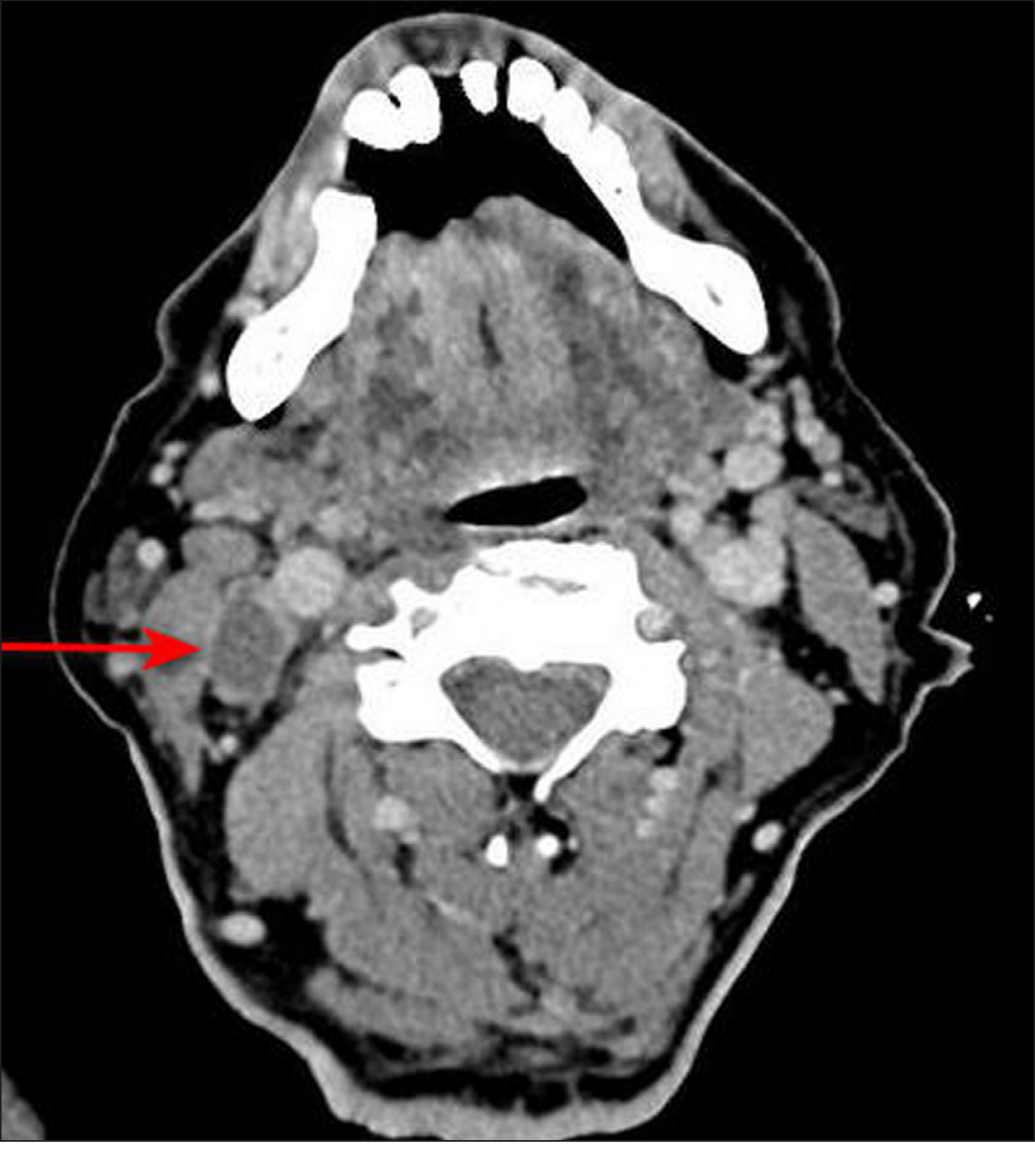


The diagnosis was confirmed by a CECT of the neck showing thrombophlebitis of the right internal jugular vein (***Figure 2***) and associated cervical necrotic lymphadenopathies.

**Figure 3:**
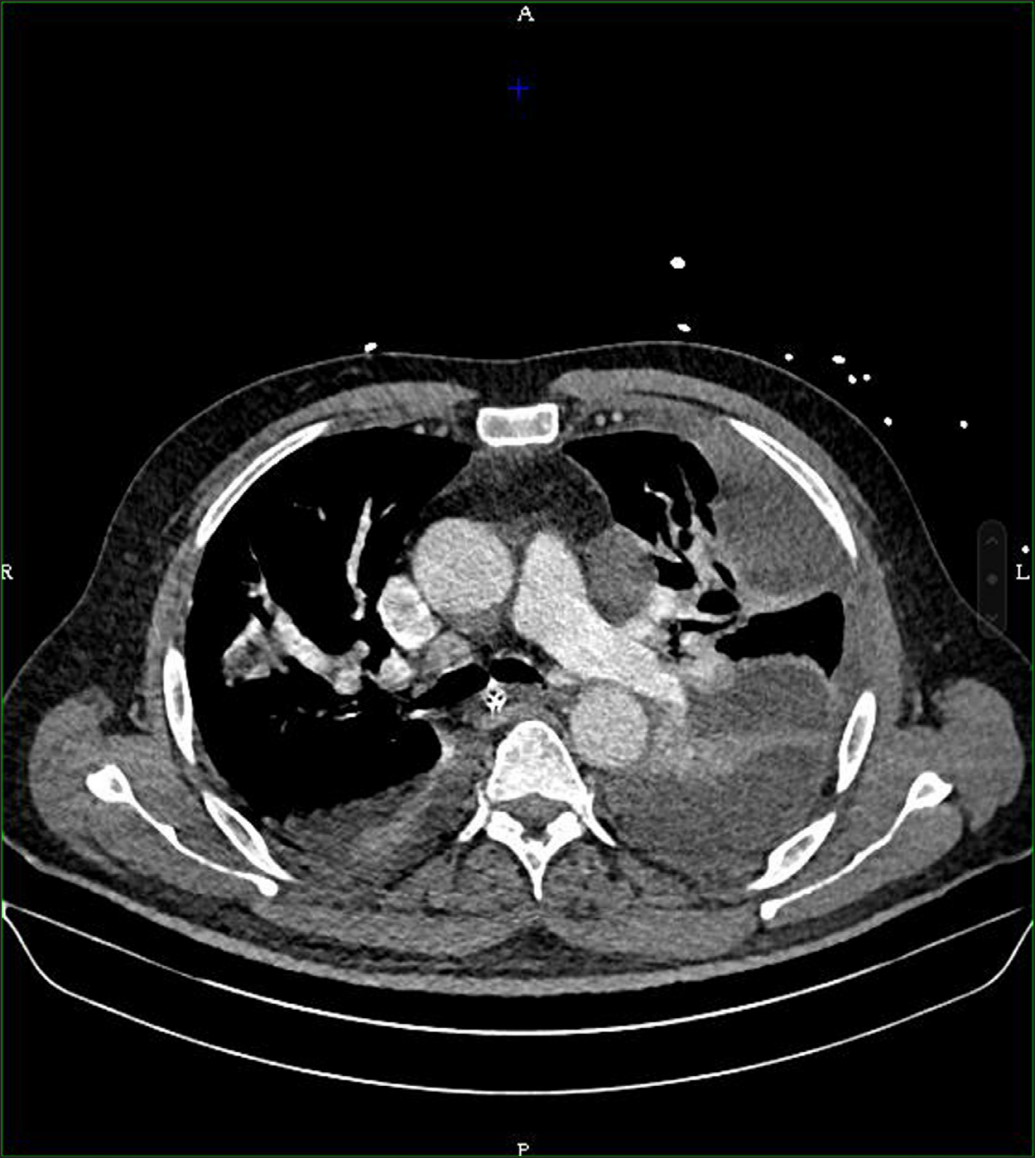


The patient was initially managed in intensive care unit. Symptoms worsened with progression of pulmonary abscesses and the development of a left empyema (***Figure 3***).

Blood culture and lymph node surgery revealed the presence of E. Coli.

After pleural drainage and a well-conducted IV antibiotic therapy, the clinical evolution was favorable, with full regression of the pulmonary lesions and complete repermeabilization of jugular vein.

## Comment

Lemierre syndrome, or post-anginal septicemia, is a rare complication of bacterial pharyngitis/tonsillitis (in 90%) leading to pulmonary abscesses via thrombophlebitis of the internal jugular vein. It generally occurs in teenagers and young adults (15–24 years old) and is rare over 40 years of age. This syndrome, common in the preantibiotic era, is seen more frequently nowadays due to antibiotic resistance. Its mortality rate remains high (4–12%) [1]. Fusobacterium necrophorum is the most frequent bacteria involved in the syndrome (80%); other agents such as Escherichia species can be involved as well. Other septic emboli locations can occur too, affecting joints, brain, liver, and kidneys.

In Lemierre syndrome, lung abscesses are secondary abscess due to hematogenous spread.

Septic pulmonary emboli may arise from other origin than cervical in Lemierre syndrome, such as right-sided endocarditis, infection elsewhere in the body with an associated septal defect, infected lines, periodontal disease, or IV drug use.

Radiographics features of septic pulmonary emboli are feeding vessel sign, associated pulmonary infacts, lower zone predilection, and lung abscesses.

Usually, the radiological triad pharyngitis, cervical vein thrombosis, and cavitating pulmonary lesions are typical of this syndrome. Thus, the radiologist may be the first to recognize it and has a key role in this still high mortality pathology.
